# Longitudinal and multi-tissue molecular diagnostics track somatic *BRCA2* reversion mutations that correct the open reading frame of germline alteration upon clinical relapse

**DOI:** 10.1038/s41525-021-00181-0

**Published:** 2021-02-22

**Authors:** Shelly Sorrells, Kelly E. McKinnon, Ashleigh McBratney, Christopher Sumey

**Affiliations:** 1 Tempus Laboratories, Chicago, IL USA; 2 Sanford Cancer Center, Sioux Falls, SD USA

**Keywords:** Cancer genomics, Predictive markers, Molecular medicine, Tumour biomarkers

## Abstract

BRCA-mutant cancers often develop therapeutic resistance through several mechanisms. Here, we report a case of pathogenic germline *BRCA2*-driven breast cancer monitored for disease progression and acquired resistance using longitudinal multi-tissue genomic testing. Briefly, genomic testing was performed throughout the course of disease on tumor tissue from multiple sites, circulating tumor DNA from blood plasma, and matched normal tissue. Genomic analyses identified actionable variants for targeted therapies, as well as emerging resistance mutations over time. Two unique *BRCA2* somatic alterations (p.N255fs and p.D252fs) were identified upon resistance to PARP inhibitor and platinum treatment, respectively. Both alterations restored the open reading frame of the original germline alteration, likely accounting for acquired resistance. This case exemplifies the evolution of multiple subclonal *BRCA* reversion alterations over time and demonstrates the value of longitudinal multi-tissue genomic testing for monitoring disease progression, predicting measures of response, and evaluating treatment outcomes in oncology patients.

## Introduction

Individuals with pathogenic germline *BRCA1*/*BRCA2* alterations have an increased risk of breast, ovarian, pancreatic, prostate, and other cancers. Tumors that arise in these patients typically exhibit a somatic mutation, loss-of-heterozygosity (LOH), or epigenetic silencing in the wild-type *BRCA* allele, resulting in truncated or absent BRCA proteins and defective homologous recombination DNA repair^1^. This homologous recombination deficiency (HRD) renders DNA particularly vulnerable to damage caused by double-strand breaks, resulting in an accumulation of mutations over time and increased carcinogenesis^2^. However, HRD also renders BRCA1/2-mutant cancers sensitive to DNA-damaging agents, such as radiation^3,4^, platinum-based therapies^5,6^, and poly ADP-ribose polymerase (PARP) inhibitors^7,8^.

PARP inhibitors target the highly abundant proteins PARP1 and PARP2, which play an important role in transcription, chromatin modification, and DNA repair^9^. Therefore, PARP inhibition targets DNA repair through multiple mechanisms of action, including PARP trapping^10,11^, inhibition of base excision repair of single-strand breaks^8^, and indirect activation of non-homologous end-joining^12–14^. In tumors with HRD, such as those with BRCA alterations, PARP inhibition is especially effective because multiple DNA repair pathways are simultaneously impaired, resulting in synthetic lethality^6–8^.

In BRCA-mutant breast cancers, single-agent PARP inhibitor treatment induces partial response rates in as high as 47% of patients, and complete response lasting 60 weeks in up to 33% of patients^15–17^. Recent studies suggest response rates continue to improve with combined treatment regimens. However, despite initial effectiveness, BRCA-mutant cancers often develop resistance to PARP inhibition^18,19^. While many potential mechanisms for this resistance have been described, *BRCA* reversion mutations have emerged as a key resistance mechanism and have been described in a number of recent cases^20–23^. *BRCA* reversions occur when acquired somatic mutations, typically insertions/deletions (indels) or base substitutions, restore the open reading frame of the altered *BRCA* allele, resulting in a functional protein that restores efficient homologous recombination DNA repair. As a result, PARP inhibition no longer causes synthetic lethality, leading to drug resistance and disease progression.

Here we report the case of a patient with pathogenic germline *BRCA2*-driven breast cancer who acquired resistance to the PARP inhibitor olaparib. The resistance likely resulted from an acquired somatic reversion mutation, which was detected by matched tumor-normal genomic analysis. A second reversion mutation was also detected after carboplatin treatment through genetic sequencing of circulating tumor DNA (ctDNA) in blood plasma. This case highlights the benefits of longitudinal genomic testing, using multiple assay and tissue types including both tissue and blood plasma, to track the evolution of tumor mutations in order to provide the best treatment options for each patient based on their unique genomic profile.

## Results

### Patient history

**Fig. 1:**
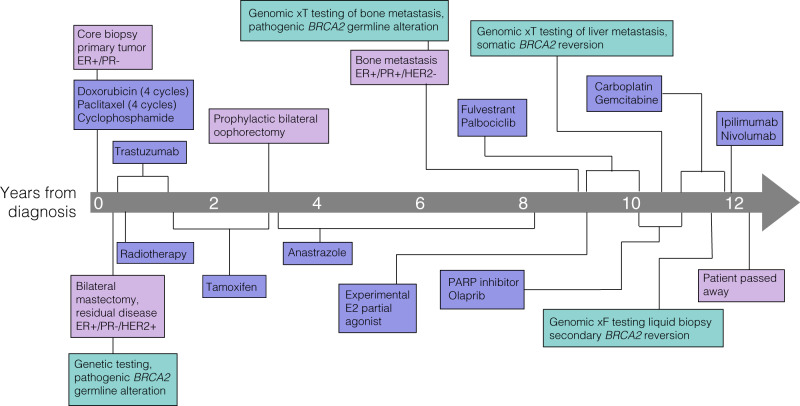
Timeline of patient procedures, treatments, and disease progression. Green boxes denote genomic testing, purple boxes denote treatments, and pink boxes denote clinical timepoints and diagnostic testing.

At the age of 50, a female patient without regular mammography screening presented with a mass in her left breast (patient timeline shown in Fig. 1). Core biopsy of the mass demonstrated invasive ductal carcinoma that was ER+, PR−, and HER2− (immunohistochemistry (IHC), 1+). Based on the size of the tumor and evidence of lymph node involvement in magnetic resonance imaging (MRI), she received neoadjuvant chemotherapy including four cycles of doxorubicin and cyclophosphamide, followed by four cycles of paclitaxel. The patient then underwent bilateral mastectomy. Pathologic analysis of the left breast demonstrated 2.5 cm of residual malignancy, which was again found to be ER+, PR−. HER2 was 2+ (IHC) with HER2 ratio at 2.5 (fluorescence in situ hybridization (FISH)). At this time the patient started adjuvant tamoxifen treatment and completed a 1 year course of trastuzumab without complication. Germline testing revealed a pathogenic *BRCA2* germline alteration, and the patient opted for a bilateral oophorectomy 2 years after starting tamoxifen. Her anti-estrogen therapy was changed to anastrozole, which she maintained for an additional 5 years. The patient palpated a mass in right axilla, but imaging workup did not show definitive evidence of malignancy or target for biopsy (mammogram, breast ultrasound, MRI, and positron emission tomography/computed tomography (PET/CT)).

One year after cessation of anti-estrogen therapy and negative imaging workup, the patient developed pain in the right breast chest wall. Chest CT identified a lesion on the sternum and subsequent PET/CT demonstrated numerous bone metastases. A dominant lesion on the sternum was biopsied and demonstrated ductal carcinoma that was ER+, PR+ and HER2 1+ (IHC, FISH, respectively). Patient timeline is presented in Fig. 1.

### Next-generation sequencing of metastatic bone lesion

**Fig. 2:**
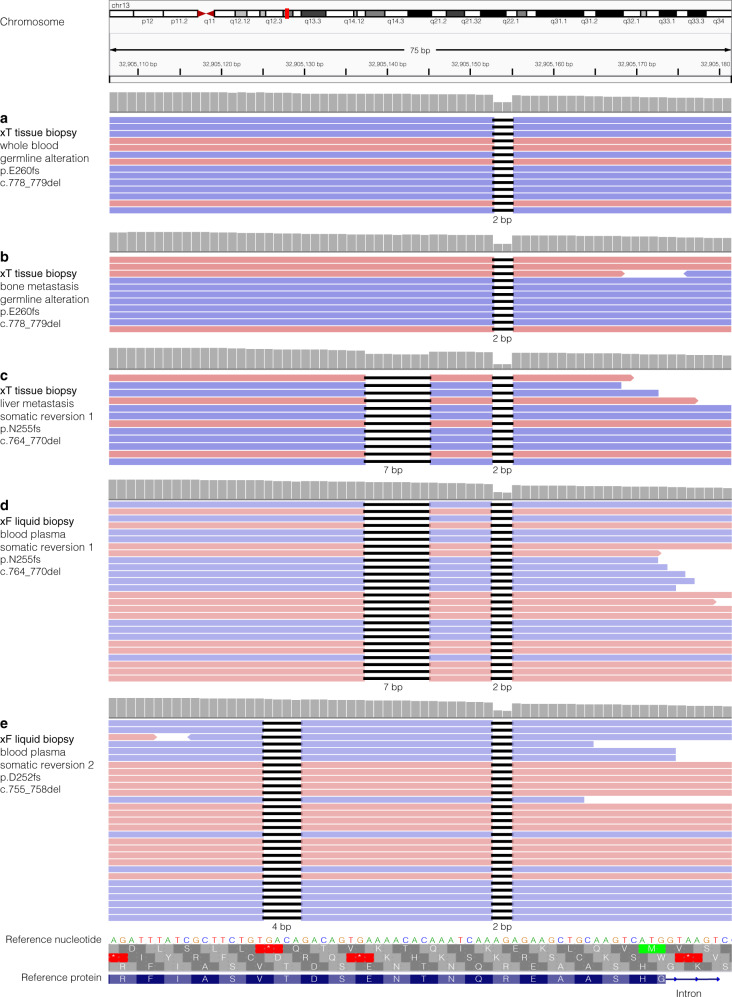
Evolution of *BRCA2* alterations over time. Integrative Genomics Viewer (IGV) visualization of BRCA2 sequencing data. **a, b** Genomic analysis of whole blood and tumor tissue from a bone metastasis reveals a 2 base pair (bp) deletion in both, indicating a germline alteration. **c** Genomic analysis of a metastatic liver lesion reveals both the original 2 bp deletion, as well as an additional 7 bp deletion, resulting in an in-frame somatic reversion. **d, e** Genomic analysis of circulating tumor DNA (ctDNA) from blood plasma shows the previously identified germline alteration and somatic reversion alteration, as well as a secondary somatic reversion mutation. Horizontal pink and blue bars are individual reads denoting forward and reverse strand sequence orientation, respectively. Gray histogram indicates relative sequencing coverage at the individual nucleotide position. A decrease in coverage is expected at the location of the deletions. Nucleotide deletions are represented as short horizontal black bars with the size of the deletion specified below the sequencing reads. Reference nucleotide sequence is indicated. Reference protein sequence at the bottom is in blue with single letter abbreviations for the amino acids and the intron depicted with a thin blue line and arrows showing directionality. The three possible open reading frames are in gray, with the third being the wild-type reading frame.

At the time of the initial cancer diagnosis, next-generation sequencing (NGS) was not part of a typical clinical workup. Due to a difference in 10 years between primary diagnosis and metastatic disease, with evidence of clinical utility and a change in hormone status, the metastatic lesion was sent for NGS sequencing. The tumor-normal matched genomic analysis of the metastatic bone lesion and blood sample confirmed the presence of the known germline *BRCA2* alteration (p.E260fs, c.778_779del, ClinVar variation ID 38119, Fig. 2a, b). Somatic loss-of-heterozygosity in *BRCA2* was not detected in sequencing results of the metastatic bone lesion. In addition to the *BRCA2* alteration, copy number gains in *CDK4* and *MYC* were also identified. The patient was treated with fulvestrant and palbociclib for 1 year, at which time she developed progression. She was briefly treated with an experimental estrogen partial agonist, but progressed shortly after. At this point, the patient began treatment with PARP inhibitor olaparib, based on the germline *BRCA2* alteration.

### Next-generation sequencing of metastatic liver lesion reveals *BRCA2* reversion mutation after clinical resistance to PARP inhibition

**Fig. 3:**
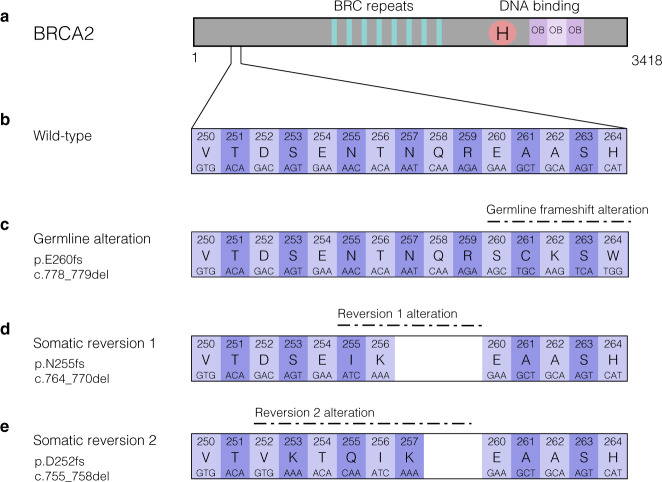
Multiple *BRCA2* reversion mutations restore open reading frame of germline alteration. **a** The BRCA2 protein diagram, protein domains, and number of amino acids. **b** The wild-type nucleotides and amino acids for amino acids 250–264, encompassing the region of germline and somatic alterations. **c** A 2 base pair (bp) deletion causes a frameshift in BRCA2 in the germline. **d** A somatic deletion of 7 bp causes a reversion mutation that restores the open reading frame of the germline alteration. **e** A 4 bp deletion causes a second somatic reversion mutation, which also restores the open reading frame altered by the germline alteration.

While the patient initially responded well to PARP inhibition, she developed liver metastases after 9 months. Genomic analysis of a metastatic liver lesion revealed the original germline alteration. A somatic alteration was also detected in *BRCA2* at a variant allele frequency (VAF) of 18.1%, which was in *cis* (on the same allele) with the pathogenic germline alteration. This somatic alteration (p.N255fs, c.764_770del) in combination with the germline frameshift resulted in an in-frame indel and subsequent restoration of the *BRCA2* reading frame (p.N255_R259delinsIK, c.764_776delinsTCAA), likely accounting for the resistance to PARP inhibition (Figs 2c and 3). The patient was then started on carboplatin and gemcitabine with excellent response in liver metastases and continued on maintenance therapy.

### Next-generation sequencing of circulating tumor DNA reveals second unique *BRCA2* reversion mutation

A liquid biopsy from blood plasma was obtained 5 months later for ctDNA sequencing. The genomic analysis identified the known somatic and germline *BRCA2* alterations (1% and 53.4% VAF, respectively), as well as an additional unique somatic *BRCA2* alteration (p.D252fs, c.755_758del) at 0.9% VAF. The secondary somatic *BRCA2* mutation was also in *cis* with the pathogenic germline alteration, but in *trans* with the first somatic reversion mutation. The second somatic mutation in combination with the germline frameshift resulted in an in-frame indel and thus represented a second subclonal reversion mutation (Figs 2d, e and 3). Additionally, the liquid biopsy revealed pathogenic variants in *ESR1* (p.Y537S, c.1610A > C) and *TP53* (p.G266V, c.797 G > T). Due to elevated tumor mutational burden (TMB) in the patient’s first sequencing results, and the possibility that previous therapies may have contributed to the development of neoantigens, the patient briefly underwent immunotherapy with one cycle of ipilimumab and nivolumab therapy. However, liver function worsened and so the therapy was discontinued. Shortly after discontinuation, the patient passed away at the age of 63.

## Discussion

Here we report a case of longitudinal diagnostic testing methodology using multiple assay and tissue types. This patient acquired resistance to PARP inhibitor olaparib, a result of a somatic *BRCA2* reversion mutation that restored the open reading frame of a germline frameshift alteration. This case is consistent with recent reports of *BRCA* reversions in both germline and somatic alterations reported in prostate cancer, ovarian cancer, and breast cancer after treatment with PARP inhibitors, which were associated with resistance to treatment^20–22,24,25^. However, in most studies to date, the timing and mechanism of the reversion alterations remain unclear due to a lack of longitudinal testing that started before the reversion alteration appeared. Indeed, many patients receive different lines of treatment or neoadjuvant therapies before PARP inhibitor treatment that may induce the accumulation of mutations^26,27^. It is possible, a small population of a clone with a reversion mutation could exist in a patient for years, and once PARP inhibition is initiated that clone is selected for and becomes the dominant clone.

This case exemplifies the evolution of multiple subclonal *BRCA* reversion alterations over time and highlights the utility of combined tissue/normal/blood biopsies in routine care of patients with cancer. For example, genomic analysis of tumor-normal matched samples enables a more thorough understanding of germline and somatic alterations and can identify co-existing actionable variants that may have been overlooked in standard genetic tests. In addition to the previously identified germline *BRCA* alteration in this case, *CDK4* and *MYC* copy number alterations were identified by NGS of matched tumor-normal tissue, informing subsequent treatment decisions. Indeed, CDK4 inhibition with palcociclib allowed for a year of successful treatment before treatment with PARP inhibitor olaparib began.

Like many patients with BRCA-mutant cancers, this patient initially responded favorably to PARP inhibition. However, after 9 months of PARP inhibitor therapy, the patient was diagnosed with progressive disease and metastasis to the liver. Genomic analysis of the liver metastasis revealed a somatic reversion mutation absent from the previous bone metastasis biopsy. This somatic reversion restored the open reading frame in tumor cells, enabling the synthesis of an in-frame BRCA2 protein and efficient DNA repair through homologous recombination, which is consistent with the resistance to PARP inhibitor treatment this patient experienced.

Additionally, this patient received a liquid biopsy that identified a second reversion alteration several months after identification of the first somatic reversion in a solid tumor biopsy. It is unclear whether the second subclonal mutation was present in another tumor site when the first reversion was identified, if it was acquired after discontinuation of PARP inhibition, or in response to the platinum treatment as has been previously documented^24,28^. While reliable at identifying driver alterations in solid tumors, due to the heterogeneous nature of tumors and emerging resistance mutations, genomic solid tissue analysis can be somewhat limited in detecting the diversity of mutations associated with advanced cancers. Indeed, many studies have identified actionable mutations in metastatic lesions that were not present in the primary tumors^29–31^. Because tumor cells from multiple locations can shed DNA into the blood, liquid biopsies can detect alterations present in distant metastases. As such, analysis of ctDNA from liquid biopsies is increasingly used in combination with solid tissue analyses.

Critical questions remain in the treatment of BRCA-mutant cancers. For example, for which patients should serial genomic testing be performed, and how should reversion alterations impact clinical decision-making? While ideally all patients would have access to serial genomic testing, in reality this is unlikely to be feasible in the near future due to cost and availability. However, serial testing will be particularly relevant for patients taking drugs with known resistance mechanisms, such as PARP inhibitors and platinum-based therapies. How reversion alterations may affect clinical decision-making likely depends on whether the reversion was detected in the tumor tissue or in blood plasma (liquid biopsy). For example, if a reversion alteration is detected through liquid biopsy, but not in the tumor tissue biopsy, continuation of PARPi treatment seems reasonable. However, the patient would benefit from being more closely monitored, as the identification of the reversion alteration in cfDNA indicates imminent progression.

In conclusion, serial NGS sequencing with multiple assays and sample types, including paired solid tumor/normal and liquid biopsy, revealed the evolution of *BRCA* reversions, the genetic source of resistance, as well as additional actionable variants for targeted therapy. This demonstrates the value of routine genomic testing in clinical care of oncology patients for monitoring disease progression, predicting measures of response, and evaluating treatment outcomes.

## Methods

### xT sample processing and nucleic acid extraction

Overall tumor content and percent tumor cellularity as a ratio of tumor-to-normal nuclei verified specimens met a 20% threshold. Solid tumor total nucleic acid was extracted from FFPE tissue sections using Chemagic 360 sample-specific extraction kits (Perkin Elmer) and digested by proteinase K.

### xT panel DNA library construction and sequencing

DNA sequencing of 596 genes was performed as previously described^32,33^. Briefly, 100 ng of DNA was mechanically sheared to an average size of 200 base pair (bp) using a Covaris Ultrasonicator. KAPA Hyper Prep Kit was used to prepare DNA libraries, which were then hybridized to the xT probe set, and amplified with the KAPA HiFi HotStart ReadyMix. Library preps were hybridized to xGEN Exome Research Panel v1.0 (Integrated DNA Technologies) and target recovery was performed using Streptavidin-coated beads. This was followed by amplification with the KAPA HiFi Library Amplification Kit. The amplified target-captured library was sequenced using 2 × 126 bp paired-end (PE) reads to an average unique on-target depth of 500× (tumor) and 150× (normal) on an Illumina HiSeq 4000. Samples were evaluated for uniformity and verified to have 95% of all targeted bp sequenced to a minimum depth of 300×.

### xF sample processing and nucleic acid extraction

Whole blood samples were collected in Streck Cell-Free DNA BCT (blood collection tubes) and centrifuged to separate plasma and buffy coat. Plasma was centrifuged a second time to remove any cellular or platelet carryovers. Plasma hemolysis was scored and reviewed by a pathologist prior to cfDNA extraction. cfDNA was isolated using the Qiagen QIAamp MinElute ccfDNA Midi Kit and the QIAcube system. The Fragment Analyzer was used to evaluate the quality of the extracted cfDNA. Quality was verified by the presence of a primary peak at approximately 150 bp and minimal to no genomic DNA (gDNA) peaks.

### xF panel cfDNA library construction and sequencing

The xF assay is a 105-gene hybrid capture NGS panel designed to detect actionable oncologic targets in plasma. Briefly, a minimum of 30 ng of cfDNA is required as input for library preparation. cfDNA libraries were prepared using the New England BioLab’s NEBNext^®^ Ultra™ II DNA Library Prep Kit for Illumina^®^, hybridized to the xF probe set, captured using Streptavidin-coated beads, and amplified with the KAPA HiFi Library Amplification Kit. The amplified target-captured cfDNA library was sequenced using 2 × 151 bp PE reads to an average unique on-target depth of 4500× on an Illumina NovaSeq 6000.

### Variant detection, visualization, and reporting

Variant detection, visualization, and reporting were performed as previously described^32,33^. Briefly, adapter-trimmed FASTQ files were aligned to the 19th edition of the human reference genome build (hg19) using Burrows-Wheeler Aligner (BWA). Following alignment, reads were grouped by alignment position and UMI family, and collapsed into consensus sequences using fgbio tools. SNV and indel variants were detected using VarDict. Copy number variants (CNVs) were analyzed using CNVkit15 plus a Tempus CNV annotation and filtering algorithm. Rearrangements were detected using the SpeedSeq analysis pipeline. Gene rearrangements were analyzed by LUMPY. Data were visualized using Integrative Genomics Viewer (IGV)^34^. Individual reads in IGV were colored pink or blue, based on forward or reverse orientation, respectively. Reads containing the deletion variants were sorted to the top for visualization and *cis* and *trans* calls were made manually.

### Ethics statement

We have complied with all ethical regulations. This is a case report of one patient, and she did not receive any experimental procedures or treatments as part of this study. As this is a retrospective analysis of data obtained through standard treatment protocols, this case does not require IRB approval. Written informed patient consent for clinical testing, analysis, and publication was obtained by Tempus Laboratories.

### Reporting summary

Further information on research design is available in the Nature Research Reporting Summary linked to this article.

## Supplementary information

Reporting Summary

## Data Availability

All relevant data supporting the findings of this study are included in the published article.
